# New insights into myosin phosphorylation during cyclic nucleotide-mediated smooth muscle relaxation

**DOI:** 10.1007/s10974-012-9306-9

**Published:** 2012-06-19

**Authors:** Sandra Puetz, Mechthild M. Schroeter, Heike Piechura, Lena Reimann, Mona S. Hunger, Lubomir T. Lubomirov, Doris Metzler, Bettina Warscheid, Gabriele Pfitzer

**Affiliations:** 1 Institute of Vegetative Physiology, University of Cologne, Robert-Koch-Str. 39, 50931 Cologne, Germany; 2 Faculty of Biology and BIOSS Centre for Biological Signalling Studies, University of Freiburg, 79104 Freiburg, Germany; 3 Clinics for Anesthesiology and Surgical Intensive Care, University of Cologne, Cologne, Germany

**Keywords:** Myosin light chain phosphorylation, Smooth muscle relaxation, cAMP, cGMP, Murine gastric fundus, NANC neurons, NO

## Abstract

Nitrovasodilators and agonists, via an increase in intracellular cyclic nucleotide levels, can induce smooth muscle relaxation without a concomitant decrease in phosphorylation of the regulatory light chains (RLC) of myosin. However, since cyclic nucleotide-induced relaxation is associated with a decrease in intracellular [Ca^2+^], and hence, a decreased activity of MLCK, we tested the hypothesis that the site responsible for the elevated RLC phosphorylation is not Ser19. Smooth muscle strips from gastric fundus were isometrically contracted with ET-1 which induced an increase in monophosphorylation from 9 ± 1 % under resting conditions (PSS) to 36 ± 1 % determined with 2D-PAGE. Electric field stimulation induced a rapid, largely NO-mediated relaxation with a half time of 8 s, which was associated with an initial decline in RLC phosphorylation to 18 % within 2 s and a rebound to 34 % after 30 s whereas relaxation was sustained. In contrast, phosphorylation of RLC at Ser19 probed with phosphospecific antibodies declined in parallel with force. LC/MS and western blot analysis with phosphospecific antibodies against monophosphorylated Thr18 indicate that Thr18 is significantly monophosphorylated during sustained relaxation. We therefore suggest that (i) monophosphorylation of Thr18 rather than Ser19 is responsible for the phosphorylation rebound during sustained EFS-induced relaxation of mouse gastric fundus, and (ii) that relaxation can be ascribed to dephosphorylation of Ser19, the site considered to be responsible for regulation of smooth muscle tone.

## Introduction

There is general consent that phosphorylation of the 20 kDa regulatory light chain (RLC) of myosin at Ser19 is a prerequisite for the actin activation of MgATPase activity of smooth muscle myosin (reviewed in Kamm and Stull 1985; Pfitzer 2001; Somlyo and Somlyo 2003). RLC phosphorylation is regulated by the balance of two opposing enzymes, the Ca^2+^–calmodulin-activated MLCK and MLCP, a type 1 phosphatase. Structural studies suggest that the actin-activated MgATPase activity of unphosphorylated myosin is low because of an asymmetric interaction between the two myosin heads (Sellers and Knight 2007). This inhibitory interaction is relieved by phosphorylation of RLC at Ser19.

Kate and Michael Bárány were among the first who carried the idea that activation of smooth muscle myosin requires phosphorylation of RLC to smooth muscle tissue. In two seminal reports, they showed that contractions elicited by norepinephrine and K^+^ in ^32^P labelled intact carotid arteries were associated with a concomitant increase in RLC phosphorylation (Barron et al. 1979) which is reversed upon relaxation (Barron et al. 1980). These proof-of-concept yet correlative studies were complemented by skinned fibre experiments in which a contraction could be elicited in the absence of Ca^2+^ with a constitutive active fragment of MLCK showing that RLC phosphorylation is sufficient to induce a contraction (Walsh et al. 1982). Subsequently, many laboratories showed that contractions elicited by a variety of agonists in different types of smooth muscle from different species are associated with an increase in RLC phosphorylation (reviewed in Arner and Pfitzer 1999; Kamm and Stull 1985; Kim et al. 2008; Somlyo and Somlyo 2003).

However, this simple concept was soon challenged by the observations of the laboratory of Murphy (Dillon et al. 1981). They found in tonic vascular smooth muscle that RLC phosphorylation was transient, i.e. it rose initially during the rising phase of the contraction but declined to lower yet suprabasal values during the sustained phase. This finding was confirmed by many other laboratories in a wide range of smooth muscle tissues and contractile stimuli (summarized in Bárány and Bárány 1996b; Kamm and Stull 1985). Interestingly, shortening velocity declined concomitant with the decline in phosphorylation suggesting that the cross-bridge cycling rate declines during a sustained contraction, i.e. the so called latch state (Dillon et al. 1981). Although several models have been put forward to account for the latch state it is still a matter of debate how it is regulated (Hai and Murphy 1989; Somlyo et al. 1988; Butler and Siegman 1998; Vyas et al. 1994; Pfitzer et al. 2005).

In keeping with the phosphorylation theory of smooth muscle contraction, relaxation of different types of smooth muscle induced by washout of the contractile agent or spontaneous relaxation of rhythmically active smooth muscle was associated with concomitant dephosphorylation of RLC (e.g. Gerthoffer and Murphy 1983; Driska et al. 1989; reviewed in Bárány and Bárány 1996b). Similarly, dephosphorylation of RLC paralleled forskolin-induced relaxation of precontracted tracheal smooth muscle (de Lanerolle 1988). Direct evidence that dephosphorylation of RLC initiates relaxation was obtained in skinned fibres incubated with a myosin phosphatase (e.g. Bialojan et al. 1987; Shirazi et al. 1994; reviewed in Hartshorne et al. 1998).

However, matters again are more complex for two reasons: (i) the rate of relaxation can be accelerated even at basal or near basal levels of RLC phosphorylation (Fischer and Pfitzer 1989; Gerthoffer et al. 1984), and (ii) in different types of tonic smooth muscles, relaxation could occur without dephosphorylation of RLC (Bárány and Bárány 1993; Gerthoffer 1986; Ishibashi et al. 1995; Katoch 1992; Katoch et al. 1997; McDaniel et al. 1992; Rembold et al. 2001; Steusloff et al. 1995; Tansey et al. 1990). Time course measurement in NO-relaxed arteries indicated that RLC are dephosphorylated during the initial phase of the relaxation but rebound to the level before addition of NO during maintained relaxation (Rembold et al. 2001; Kitazawa et al. 2009). These findings gave rise to the hypothesis that contraction may be turned off independent of RLC dephosphorylation by additional regulatory mechanisms (Brophy et al. 1999; Rembold et al. 2000; Pfitzer et al. 1993).

The uncoupling between relaxation and RLC dephosphorylation was particularly evident when relaxation was actively induced by agents that increase intracellular cGMP (McDaniel et al. 1992; Rembold et al. 2001; Kitazawa et al. 2009) or cAMP levels (Bárány and Bárány 1993). Although this uncoupling is the apparently most striking feature, we consider the observation that RLC phosphorylation remains elevated, the even more puzzling phenomenon. This is because two processes should synergistically favour dephosphorylation of RLC: (i) MLCK should be inactive due to a decrease in cytosolic [Ca^2+^] (DeFeo and Morgan 1989; McDaniel et al. 1992), and (ii) cGMP and cAMP enhance the activity of MLCP (Etter et al. 2001; Kitazawa et al. 2009; Lubomirov et al. 2006). Therefore, we hypothesized that Ser19, which is predominantly phosphorylated by MLCK and is associated with activation of smooth muscle contraction, is in fact dephosphorylated during relaxation and that the phosphorylation rebound is due to phosphorylation of one of the other sites. The other sites include Ser1, Ser2, Thr9 and Thr18. Thr18 is phosphorylated by MLCK albeit at a much lower rate than Ser19 whereby phosphorylation occurs at random, i.e. phosphorylation of Ser19 is not a prerequisite for Thr18 phosphorylation (Bresnick et al. 1995). Thr18 and Ser19 are phosphorylated with equal efficiency by integrin-linked kinase, ILK (Wilson et al. 2005), and ZIP kinase (Niiro and Ikebe 2001; Walsh 2011 for review). Ser1 and 2, Thr9 are phosphorylated by PKC (Bengur et al. 1987). Phosphorylation of Thr 9 has been suggested to be involved in the relaxant effect of okadaic acid in basilar arteries (Obara et al. 2008).

To test the hypothesis, that phosphorylation of Ser19 does not account for the uncoupling of stress from RLC dephosphorylation, we chose smooth muscle from gastric fundus as a model. This preparation can be relaxed by electric field stimulation (EFS) within seconds. It is well established that EFS elicits relaxation by activating non-adrenergic non-cholinergic (NANC) neurons of the plexus myentericus which in turn release NO and vasointestinal peptide (VIP) leading to an increase in cGMP and cAMP levels in the fundus smooth muscle cells (reviewed in Lefebvre et al. 1995). An advantage of this preparation is that relaxation is not diffusion limited which is different from vascular smooth muscle, in which relaxation is induced by NO-donors or agonists. Furthermore, relaxation occurs within seconds compared to minutes in the vascular tissue allowing us to determine whether uncoupling of relaxation from dephosphorylation is independent of the time scale at which relaxation occurs.

## Materials and methods

### Materials

The used primary antibodies were rabbit polyclonal anti-phospho-myosin light chain Ser-19 (pSer19), Rockland, PA, USA; rabbit polyclonal anti-phospho-MLC20 Thr-18 (pThr18), Santa Cruz, CA, USA; anti-phospho-myosin light chain 2 pThr18/pSer19, Pierce Biotechnology, Rockford, IL, USA; and mouse monoclonal anti-MLC20 antibody for total MLC20 (RLC), Sigma, Germany. The secondary antibodies were IRDye conjugated goat anti-mouse (IRDye680) and goat anti-rabbit (IRDye800), LI-COR, NE, USA and horse radish peroxidase-conjugated donkey anti-mouse and donkey anti-rabbit both from Dianova, Germany. Thermolysin came from R&D Systems, Minneapolis, USA. The Sypro^®^ Ruby, Silver Snap^®^ stain and Bio-Rad Protein Assay^®^, were from Bio-Rad Laboratories GmbH, Germany; the enhanced chemiluminescence Kit (Pierce ECL Western Blotting Substrate) from Perbio Science/Thermo Fisher Scientific, Germany; and the Phos-tag kit from Wako Chemicals GmbH, Germany. All other chemicals were from Sigma or AppliChem, Germany.

### Muscle strip preparation

Male mice, 12 weeks old, were killed by cervical dislocation following procedures approved by the Institutional Animal Care and Use Committee of the University of Cologne. The stomach was quickly removed and transferred to PSS with low calcium (in mM): 118 NaCl, 5 KCl, 1.2 Na_2_HPO_4_, 1.2 MgCl_2_, 0.16 CaCl_2_, 24 Hepes, 10 glucose, pH 7.4 at 37 °C and bubbled with 100 % O_2_. Murine gastric fundi were freed of mucosa, cut in the direction of the circular muscle layer into strips (2 mm wide) and mounted vertically in Ca^2+^-free PSS in an organ bath between two platinum electrodes. Slack length was determined in Ca^2+^–free PSS, the fundus strips were then stretched to 167 % of slack length in low calcium PSS. Calcium was slowly increased up to 1.6 mM and a control contraction was induced by depolarization with 45 mM K^+^, replacing Na^+^ in the PSS solution. Force was recorded with a type Q11 transducer (Hottinger Baldwin Messtechnik, Germany). Adrenergic/cholinergic transmission as well as prostaglandins were inhibited by addition of atropine (10 μM), propranolol (1 μM), phentolamine (1 μM) and indomethacin (1 μM) to PSS for 20 min. This inhibitor cocktail was present throughout the experiment. Contractions were elicited with 5 nM endothelin-1 (ET-1). Electrical field stimulation (EFS) was performed with custom-made stimulators with trains of pulses at 10 Hz [30 V, pulse width 0.5 ms] for 30 s. The strips were snap frozen within 100 ms with liquid nitrogen precooled tongs at distinct time points of EFS (0, 2, 5 and 30 s) and fixed in dry-ice precooled 15 %TCA/acetone.

### Determination of the intracellular Ca^2+^-transient

For the simultaneous determination of force and intracellular [Ca^2+^], the muscle strips were mounted horizontally in a myograph on the stage of an inverted Zeiss microscope (Axiovert 35, Zeiss, Jena) using the cuvette of the confocal wire myograph system 120CW (DMT, Aarhus Denmark). The strips were equilibrated as above. After the K^+^-induced test contraction, the strips were loaded with fura-2 for 4 h with 1 μM acetoxy methylester fura-2 (fura-2 AM) dissolved in DMSO premixed with pluronic FF127 (1 μM final; Lucius et al. 1998). After loading, the strips were washed 2 × 15 min with PSS to remove extracellular fura-2 AM. The fluorescence signal was recorded during the endothelin-induced contraction and EFS-induced relaxation with an imaging system from TILL Photonics (Planegg, Germany). In brief, UV-light of alternating wave lengths (340 and 380 nm) was obtained by a fast monochromator positioned in front of a high pressure xenon lamp passed with light guides and focused onto the muscle strip through the quartz window on the bottom of the cuvette. The fura-2 emission (510 nm) was passed through the objective lens (fluor ×10, Zeiss, Jena), collected with a CCD camera (TILL Photonics Imago PCO Imaging, SensiCam) and analysed using TILL vision image 3.0 software. Force was recorded with MyoDaq (DMT). Synchronization of the force and the fluorescence signal was performed with homemade software. In some experiments the fura-2 signal was calibrated according to the protocol of Himpens and Somlyo (1988). In brief, the minimal ratio, *R*_min_ was determined after incubating the strips with 10 μM ionomycin for 10 min in Ca^2+^-free calibration buffer containing ((in mM) 140 KCl, 2 EGTA, 24 HEPES, 1.2 MgCl_2_, 1.2. Na_2_HPO_4_, pH 6.8). Then 10 mM Ca^2+^ was added to obtain the maximal ratio, *R*_max_, followed by addition of 20 mM MnCl_2_ to record background values. From these values, [Ca^2+^] was calculated using the formula and an apparent dissociation constant of the Ca^2+^–fura 2 complex of 224 nM given by Grynkiewicz et al. (1985).

### Two dimensional gel electrophoresis

**Fig. 1:**
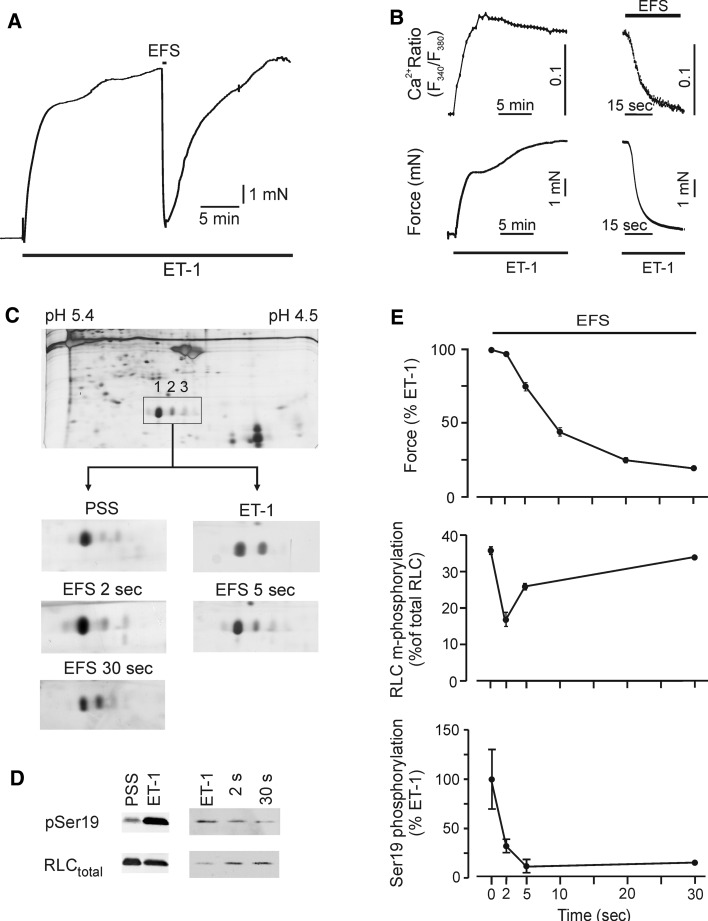
Phosphorylation transients during electrical-field-induced relaxation of endothelin-1 (ET-1) precontracted smooth muscle strips from mouse gastric fundus. **a** Representative force tracing of ET-1-induced contraction and EFS-induced relaxation (10 Hz, 0.5 ms pulses, duration 30 s) and force recovery. **b** Simultaneous determination of intracellular Ca^2+^-transients and force in ET-contracted (*left panels*) and EFS-relaxed fundus tissues. The ratios of the fluorescence signals excited by 340 nm, and 380 nm (F_340_/F_380_) were used as indicator of intracellular [Ca^2+^]; representative tracings from 6 independent experiments. **c** Determination of RLC phosphorylation by 2D-PAGE; *spot 1* and *2* refer to, respectively, unphosphorylated and monophosphorylated RLC. The intensity of *spot 3* amounts to ~14 % of total RLC and does not change significantly in contracted and relaxed preparations. *PSS* resting conditions prior to ET-1 stimulation, *ET-1* plateau of contraction just prior to EFS and 2, 5 and 30 s after starting EFS. **d** Western blots with pSer19 and RLC total antibody, *left panel* separation of proteins with PhosTag gels and visualization of immunoreactivity with the odyssey system; *right panel* separation by 15 % SDS-PAGE and visualization of the immunoreactivity with ECL. **e** Summary of time courses of EFS-induced relaxation (*upper panel*), m-phosphorylation of RLC determined by 2D-PAGE (*middle panel*) and m-phosphorylation of Ser19 normalized to immunoreactive signal obtained with the total RLC antibody and expressed in  % of the ET-1 value (*lower panel*). A similar result was obtained when pSer19 was expressed relative to the Coomassie stained desmin band. Symbols represent mean ± SEM of 4–9 determinations

TCA was removed from the strips by several acetone washes, afterwards the strips were air-dried. The strips were homogenized in 50-μl sample buffer (10 mM Tris–HCl, pH 7.5, 9.2 M urea, 3 % ampholines pH 4.5–5.4, 10 mM DTT and 0.0001 % bromphenol blue). The lysates were subjected to 2D-PAGE and the silver or Sypro Ruby^®^ stained gels were analysed as described using phoretix software (Biostep, Germany) (Lucius et al. 1998). Regularly 3 major spots can be resolved. It is generally accepted that the most basic spot (spot 1 in Fig. 1c) and the adjacent, more acidic spot (spot 2 in Fig. 1c) represent unphosphorylated and monophosphorylated RLC, respectively. Spot 3 is considered to represent diphosphorylated RLC and unphosphorylated non-muscle RLC (Gagelmann et al. 1984; Gaylinn et al. 1989).

### SDS-PAGE/western blotting

TCA was removed as above. For separation by SDS-PAGE, the strips were prepared and subjected to SDS-PAGE as described by Lubomirov et al. (2006). The separated proteins were transferred to nitrocellulose according to Towbin et al. (1979). To use the intensity of desmin as an additional internal loading control, the gel area with proteins of molecular masses between 40 and 60 kDa was not transferred but stained with Coomassie R-250^®^, and the desmin band was evaluated densitometrically using the Odyssey Infrared Imaging System (LI-COR).

For separation by Phos-tag gel electrophoresis, the fundus strips were agitated for 2 h in 120 μl sample buffer B (65 mM Tris–HCl pH 6.8, 4 % SDS, 100 mM DTT, 5 % glycerol, 0.04 % bromphenol blue), boiled and centrifuged as described above. The protein concentration was determined in the supernatant according to Bradford (1976) using BSA as standard. Equal amounts of protein were subjected to Phos-tag SDS-acrylamide electrophoresis and transferred to nitrocellulose as described by Takeya et al. (2008).

Nitrocellulose membranes were first probed with antibodies against pSer19, pThr18, or RLC total followed by incubation with the appropriate secondary antibody and visualization of the immunoreactive signal with enhanced chemiluminescence ECL. Blocking of non-specific binding sites and densitometric evaluation of immunoreactive bands were performed as in Lubomirov et al. (2006). When using the Odyssey system (LI-COR) for visualization of the signal, the blots were first probed with the phosphospecific antibodies (pSer19, pThr18, pTrh18/pSer19) and the appropriate IRDye conjugated secondary antibodies. Thereafter, the same blot was reprobed with a monoclonal mouse antibody against total RLC. For Odyssey imaging, membranes were blocked in 2 % milk/TBS (10 mM Tris pH 8.0, 150 mM NaCl) and probed with primary antibody in 2 % milk/TBST (TBS with 0.1 % Tween 20 (v/v)), in case of pThr18 in 1 % BSA/TBST. Detection and quantification of the infrared signals were performed using the Odyssey system software.

### Protein digestion and mass spectrometry

Following staining with colloidal Coomassie Brilliant Blue G-250, protein spots of interest were cut from the gel, destained by alternated incubation with 20 μl of 10 mM NH_4_HCO_3_ and 5 mM NH_4_HCO_3_/50 % acetonitrile (ACN) (v/v) for 10 min and dried in vacuo. In-gel digestion of proteins was performed for 2 h at 60 °C using 2 ng thermolysin dissolved in 5 μl of 50 mM NH_4_HCO_3_. Proteolytic peptides were extracted by incubating the gel spots twice with 10 μl of 95 % ACN (v/v)/0.1 % trifluoroacetic acid (TFA) (v/v) for 15 min. Extracts were combined and dried in vacuo. For mass spectrometric analysis, peptides were re-dissolved in 15 μl 0.1 % TFA (v/v).

Online reversed-phase nano-HPLC separations were performed using the UltiMate 3000 RSLC System (Dionex/Thermo Fisher, Idstein, Germany) equipped with two precolumns (Acclaim^®^ PepMap μ-Precolumn Cartridge; 0.3 mm × 5 mm, particle size 5 μm) and an Acclaim^®^ PepMap RSLC analytical column (75 μm × 25 cm, C18, 2 μm, 100 Å). Peptides were preconcentrated on the precolumn and washed for 5 min using 0.1 % (v/v) TFA at a flow rate of 30 μl/min. Subsequently, peptides were separated at a flow rate of 300 nl/min using a binary solvent system consisting of solvent A [0.1 % formic acid (v/v)] and solvent B [0.1 % FA (v/v), 84 % ACN (v/v)]. The following gradient was used: 5–40 % solvent B in 30 min and 40–95 % solvent B in 5 min. The column was then washed for 5 min with 95 % solvent B and equilibrated with 5 % solvent B for 15 min.

The LTQ Orbitrap XL instrument was equipped with a nanoelectrospray ion source (Thermo Fisher Scientific) and distal coated SilicaTips (FS360-20-10-D, New Objective, Woburn, USA). The instrument was externally calibrated using standard components. The general mass spectrometric parameters were as follows: spray voltage, 1.5 kV; capillary voltage, 45 V; capillary temperature, 200 °C; and tube lens voltage, 120 V. For data-dependent MS/MS analyses, the software XCalibur 2.0.7 (Thermo Fisher Scientific) was used. Full scan MS spectra were recorded from *m*/*z* 370 to 1700 and acquired in the Orbitrap with the Automatic Gain Control (AGC) set to 5 × 10^5^ ions and a maximum fill time of 500 ms. The five most intense multiply charged ions were selected for fragmentation by multistage activation (MSA). MSA scans were performed in the linear ion trap with an AGC set to 10,000 ions and a maximum fill time of 400 ms. Fragmentation was carried out using the following parameters: normalized collision energy, 35 %; activation q, 0.25; activation time, 30 ms; ion selection threshold, 2500; dynamic exclusion, 45s; multistage activation enabled; and listed neutral losses *m*/*z* 98, 49, 32.6.

Mass spectrometric data were processed using Mascot Deamon v. 2.3.0 and searched against the NCBI database (taxonomy filter *Mus musculus*, 143,284 sequences) using the MASCOT algorithm v. 2.3.02 (Perkins et al. 1999). For database searches, no enzyme was specified and mass tolerance was set to 5 ppm and 0.4 Da for peptide and fragment ion masses, respectively. Oxidation of methionine and phosphorylation of serine, threonine and tyrosine were considered as variable modifications. Proteins above a MASCOT significance threshold of 0.05 were considered unambiguously identified. Common contaminants (i.e. keratin) were excluded. Fragmentation spectra of phosphopeptides were manually annotated and validated.

### Statistical analysis

All data are mean ± SEM; *n* is the number of strips. Statistical significance was determined using unpaired *t* test with Welch’s correction for unequal variances or ANOVA followed by Bonferroni post test for multiple comparisons when applicable (Graph Pad software). The level of significance was set at *p* < 0.05.

## Results

### Uncoupling of cyclic nucleotide-mediated relaxation from RLC dephosphorylation

Isolated smooth muscle strips from gastric fundus responded to ET-1 with a stable tonic contraction amounting to 7.45 ± 0.44 mN (*n* = 41), and to EFS with a TTX sensitive relaxation. The relaxation amplitude increased with increasing frequencies and was maximal at 10 Hz, which relaxed the ET-1 precontracted strips by 80 ± 2 % (*n* = 16). This frequency was used throughout. The time course of relaxation was biphasic starting with a lag period of ~1.2 s followed by an exponential decay (Fig. 1e, upper panel). Half time of relaxation (*T*_1/2_) was 8.0 ± 0.13 s, and relaxation was stably maintained for at least 30 s. ET-1-induced contraction was associated with an increase in intracellular [Ca^2+^] from ~142 nM under basal conditions (PSS) to ~310 nM. Upon EFS, intracellular [Ca^2+^] decreased in parallel with relaxation and remained low during maintained relaxation (Fig. 1b).

In the next series of experiments, we investigated whether EFS-induced relaxation was associated with respective changes in phosphorylation of RLC using 2D-PAGE. As predicted by the increase in intracellular [Ca^2+^], RLC mono- (m-) phosphorylation (spot 2 in Fig. 1c) increased from 9 ± 1 % (*n* = 3) under resting conditions (PSS) to 36 ± 1 % of RLC_total_ (*n* = 4, *p* = 0.003). The time point chosen was such that it represented the phosphorylation status just preceding EFS-induced relaxation, i.e. during the tonic phase of the contraction. EFS-induced relaxation was preceded by a decline in m-phosphorylation to 18 ± 1 % of RLC_total_ (*p* = 0.004) within 2 s after starting EFS. Thereafter, RLC were rephosphorylated (Fig. 1c, e, middle panel); 30 s after starting EFS RLC phosphorylation was similar to the value of the ET-1 contracted tissue. Thus, despite the fact that relaxation as well as the decay in intracellular [Ca^2+^] was sustained, dephosphorylation of rRLC was only transient suggesting that force is uncoupled from m-phosphorylation during cyclic nucleotide-mediated relaxation. A similar observation has been made before in uterine smooth muscle relaxed by isoprenaline (Bárány and Bárány 1993) and arterial smooth muscle relaxed by NO-donors (e.g. McDaniel et al. 1992; Rembold et al. 2000).

Under resting conditions, as well as in ET-1 contracted and EFS-relaxed preparations, a third spot could be resolved in the 2D-PAGE (c.f. Fig. 1c). This spot was extensively characterized by Bárány and Bárány (1996a) and corresponds to spot 2 in their nomenclature. They proposed that it contains a mixture of diphosphorylated and non-phosphorylated, non-muscle RLC and estimated that the contribution of non-muscle RLC was between 8 and 16 % (Mougios and Bárány 1986). In our experiments, the intensity of this spot (14 % in PSS, 13.8 ± 0.4 % in ET-1 (*n* = 3) and 12 ± 1 in 30 s EFS-treated tissues (*n* = 4) of RLC_total_) did not differ significantly between the different treatments suggesting that only a small amount of RLC was di-phosphorylated. This conclusion was confirmed by western blots probed with phosphospecific antibodies directed against di-phosphorylated RLC (Thr18/Ser19), in which no immunoreactive signal was detected (c.f. Fig. 3c). We therefore focused on the m-phosphorylation in the further analysis.

It was proposed that the neurotransmitters responsible for EFS-induced relaxation involve NO and VIP and their respective downstream signals cGMP–PKG and cAMP–PKA (Lefebvre et al. 1995). Exogenous application of NO as DEA–NO (300 μM) and VIP (1 μM) relaxed the preparations by 90 ± 3 and 80 ± 5 % (*n* = 6) with a half time of ~30 s. RLC m-phosphorylation during sustained relaxation induced by DEA–NO and VIP was not significantly different from the value of ET-1 contracted fundus [DEA–NO: 28 ± 4 %, *n* = 5, *p* = 0.14 vs. ET-1 and VIP: 33 ± 4 %, *n* = 8, *p* = 0.75 vs. ET-1]. Thus, although relaxation induced by exogenously added neurotransmitters was much slower, it was also uncoupled from RLC m-phosphorylation in a similar manner as with EFS-induced relaxation irrespective of whether the neurotransmitters acted through cGMP or cAMP. NO–cGMP signalling may predominate under our conditions since l-NAME inhibited relaxation by ~75 % (data not shown), which is in line with an earlier report showing that EFS-induced relaxation in PKG knock-out mice is significantly blunted (Pfeifer et al. 1998).

### Uncoupling of force from RLC m-phosphorylation is not due to phosphorylation of Ser19

It has been implicitly assumed that m-phosphorylation is due to Ser19 phosphorylation. However, since intracellular [Ca^2+^] was low during maintained relaxation suggesting low MLCK activity, we gave consideration to the possibility that m-phosphorylation at 30 s was not caused by Ser19 phosphorylation, the major MLCK site. Indeed, western blot analysis with pSer19 phosphospecific antibodies revealed that pSer19 immunoreactivity was high in lysates from ET-1 contracted fundus strips and rapidly declined in EFS-treated preparations in parallel with relaxation (Fig. 1d, e, lower panel). During the sustained phase of relaxation pSer19 immunoreactivity was 18 ± 7 % (*p* < 0.01, *n* = 9) of the value before starting EFS in ET-1 contracted strips which was taken as 100 %. For comparison, under resting condition (PSS before addition of ET-1) it was 11 ± 4 % of ET-1 (*n* = 3). Using Phos-tag gels which allow to separate m- from di-phosphorylated phospho-species of RLC (Takeya et al. 2008), we confirmed that the antibody only detected monophosphorylated RLC (c.f. Fig. 3). These results gave rise to the interesting possibility that m-phosphorylation during sustained relaxation involves a site different from Ser19.

### Determination of the m-phosphorylated site during relaxation

**Fig. 2:**
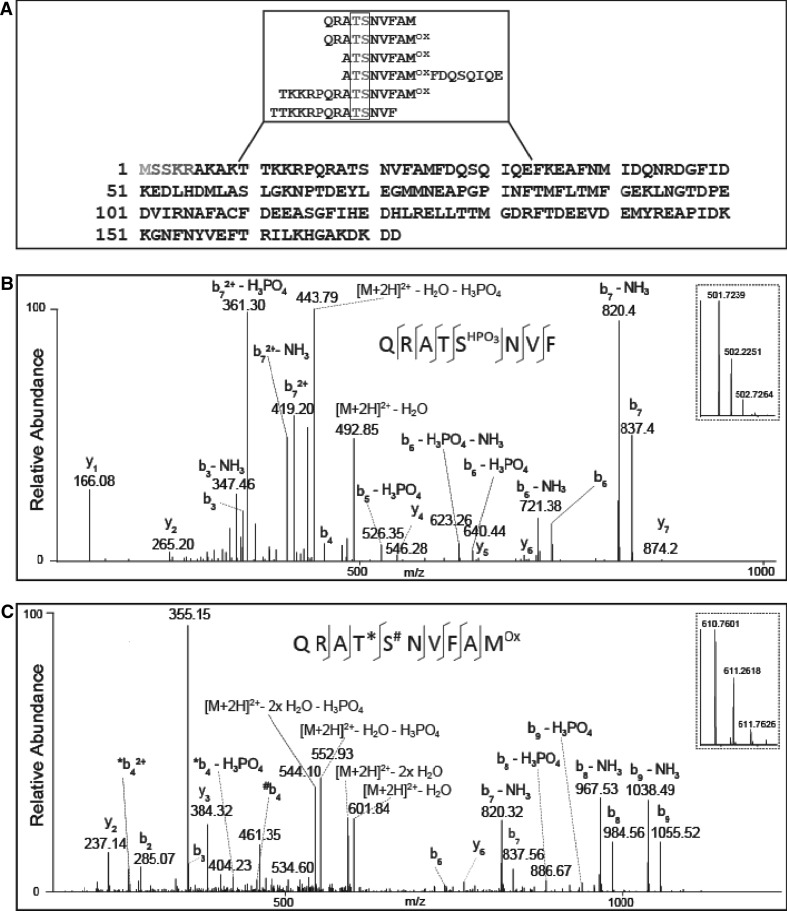
Identification of RLC and fragment spectra of phosphopeptides. **a** Sequence of RLC. Amino acid sequences identified by MS are marked in *black*. In total, a sequence coverage of 97 % was achieved. Phosphorylation of RLC at Thr18/Ser19 (*boxed gray letters*) was confirmed by multiple peptides (*inset*). **b** MS/MS spectrum of the monophosphorylated peptide QRATS*NVF (*m*/*z* 501.7239) of myosin regulatory light chain from fully contracted muscle. The phosphorylation site is localized to S19. **c** MS/MS spectrum of the monophosphorylated peptide QRATSNVFAM^Ox^ (*m*/*z* 610.7601) of myosin regulatory light chain from muscle treated with EFS for 30 s. The fragment ions observed in the spectrum support monophosphorylation events at T18 (*) as well as S19 (#). The corresponding survey scans for each peptide are displayed as zoom-in views

To determine which site of RLC is m-phosphorylated in 30 s EFS-relaxed strips, the unphosphorylated (spot 1 in Fig. 1c) and m-phosphorylated spot (spot 2 in Fig. 1c) were cut out from 2D-PAGE from ET-1 and 30 s EFS-treated samples and subjected to high resolution liquid chromatography tandem mass spectrometry (LC/MS/MS). Following digestion of protein spots with thermolysin, myosin regulatory light chain polypeptide 9 (*Mus musculus*), i.e. RLC, was unambiguously identified in all spots with a sequence coverage of 89–97 %. Furthermore, using multistage activation as an effective method for fragmentation of phosphopeptides, no phosphorylation was detected in the protein spot assigned non-phosphorylated, whereas phosphorylated peptide species were reliably detected in the spots of phosphorylated RLC from both ET-1 and 30 s EFS-treated samples. In the ET-1 treated sample, Ser19 was determined to be the major phosphorylated residue in RLC by MS/MS (Fig. 2b) confirming our results with the pSer19 antibody.

We then hypothesized that the N-terminal PKC site, Thr9, might be m-phosphorylated in EFS-treated samples because this site was found to be phosphorylated during okadaic acid-induced relaxation of smooth muscle (Obara et al. 2008) reported to occur without dephosphorylation of RLC (Tansey et al. 1990). However, no peptides were detected, which were phosphorylated at this site. Rather, m-phosphorylated peptide species of RLC were identified with evidence for Thr18 and Ser19 as the specific site of phosphorylation (Fig. 2c). These data indicate that the level of Thr18 phosphorylation was higher in EFS than ET-1 treated samples. Since these reversible phosphorylation events take place at neighbouring amino acid residues (Ser19 and Thr18), it was not possible to separate the corresponding phosphopeptide isoforms of RLC present in the m-phosphorylated 2-D gel spot. For the very N-terminus of the protein containing Ser1 and Ser2, which are phosphorylated by PKC (Bengur et al. 1987), no peptide could be detected, and hence, no evidence for further reversible phosphorylation events at these specific sites was retrieved in this work.

**Fig. 3:**
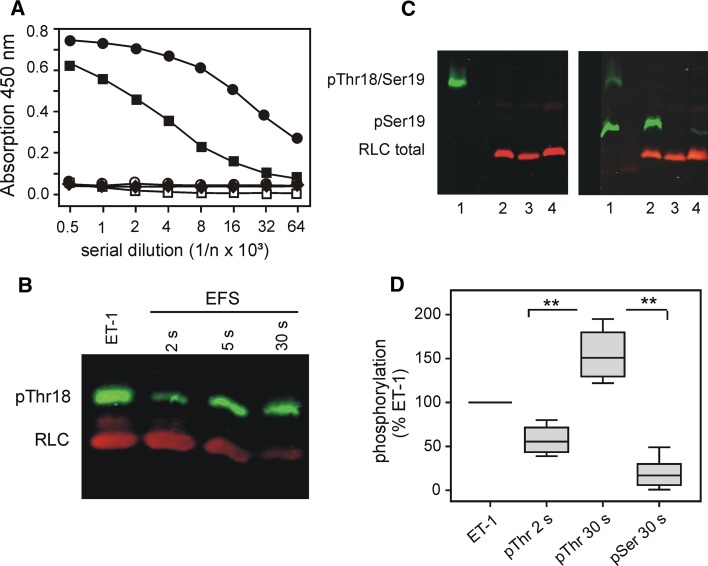
Determination of m-phosphorylation of Thr18 during EFS-induced relaxation using phosphospecific antibodies. **a** Assessing specificity of anti-phospho-antibody p-MLC (Thr18)-R (Santa Cruz Biotechnology #sc-19848R) by ELISA. As substrate a differently phosphorylated peptide identical to amino acid residues 11-26 of myosin light chain polypeptide 9 *Mus musculus* (NCBI Protein Data Bank NP_742116) was used. The antibody exhibited a higher affinity against monophosphorylated Thr18 (*closed circles*) than against diphosphorylation at Ser19 and Thr18 (*closed squares*). The immunoreactivity against m-phosphorylation at Ser19 (*closed diamonds*) and the nonphosphorylated peptide (*open circles*) was very poor and came close to the one measured against ovalbumin (*open squares*). **b** Representative western blots with phosphospecific antibodies against m-phosphorylated Thr18, and **c** pTrh18/pSer19 diphosphorylated RLC (*left panel*) and m-phosphorylated Ser19 (RLC) and total RLC. The different phosphospecies of RLC were separated by Phos-tag gels, *lane 1* mouse tail artery arteries incubated at pCa 6.8 and in the presence of 10 μM microcystin, *lane 2–4* gastric fundus contracted with ET-1 (*lane 2*), relaxed with EFS for 5 s (*lane 3*) and 30 s (*lane 4*). **d** Summary of changes of m-pThr18 during EFS-induced relaxation, pSer19 is replotted from Fig. 1. *Bars* represent box plots of *n* = 4 pThr18 and *n* = 10 pSer19 determinations, ** *p* < 0.01

Based on these results, we reasoned that m-phosphorylation of Thr18 significantly contributes to the phosphorylation rebound in the EFS-relaxed tissue. This hypothesis was tested with western blot analysis using a commercially available antibody against m-phosphorylated Thr18. While the commercially available antibody against pSer19 has been widely used, we are aware of only few studies which used this pThr18 antibody (e.g. Getz et al. 2010). Therefore, we first assessed its specificity in an ELISA assay using differently phosphorylated peptides derived from RLC (aa 11–26). Figure 3a shows that the antibody reacted neither with the non-phosphorylated nor with the Ser19 m-phosphorylated peptide whereas it recognized the Thr18 m-phosphorylated and with a much lower affinity the diphosphorylated peptide. As shown in Fig. 3b and to our surprise, immunoreactivity with this antibody in lysates from ET-1 treated samples was frequently higher than expected from our MS/MS data and from the literature (Bárány and Bárány 1996b for review). The reason for this discrepancy is not clear at present. We cannot exclude the possibility that there is some crossreactivity with pSer19 which was not detected with the short RLC peptides used in the ELISA assay. Compared to ET-1, pThr18 immunoreactivity during the initial phase of relaxation (2 and 5 s) was lower (Fig. 3b, d). The signal intensity increased again and was significantly higher in preparations relaxed for 30 s compared ET-contracted preparations (Fig. 3d). We confirmed that the rise in pThr18 immunoreactivity was not due to an increase in diphosphorylation with Phos-tag gels and the pThr18/pSer19 dual phosphorylation antibody (Fig. 3c). Taking together the results with the pSer19 and the pThr18 antibodies, we propose that rephosphorylation of RLC during sustained relaxation can be ascribed to m-phosphorylation of pThr18 whereas m-phosphorylation of pSer19 remains low.

## Discussion

Although not unequivocally found several authors reported that relaxation mediated by NO-donors and isoprenaline of different types of smooth muscle tissues from different species occurs without dephosphorylation of RLC or that dephosphorylation was only transient (reviewed in Pfitzer 2001) leading to the statement of Kate and Michael Bárány: ′it can be concluded that RLC dephosphorylation is not a prerequisite of smooth muscle relaxation′ (Bárány and Bárány 1993). Determination of RLC phosphorylation with 2D-PAGE during relaxation of gastric smooth muscle induced by activation of NANC neurons corroborates these conclusions. However, by analysing which amino acids are phosphorylated, we present evidence that this conclusion has to be modified. The novel finding of our study is that dephosphorylation of pSer19 does correlate with relaxation, and hence, this site cannot account for the rebound in RLC m-phosphorylation. Based on LC/MS and western blot analysis, we suggest that m-phosphorylation of Thr18 increases during sustained relaxation. Hence, we propose that the rebound in RLC phosphorylation is due to a redistribution of phosphorylated residues in favour of m-phosphorylation of Thr18.

Only a limited number of investigations determined the phosphorylated residues in intact tissue stimulated with different agonists using phosphopeptide mapping in combination with phosphoamino acid analysis with ^32^P. To the best of our knowledge, our study is the first to apply MS/MS analysis. According to Bárány and Bárány (1993), the m-phosphorylated spot contained pSer and pThr and suggested a pSer to pThr ratio of 6:1 (Csabina et al. 1986). Others confirmed that the major ^32^P-labelled amino acid residue was Ser (e.g. McDaniel et al. 1992; D’Angelo et al. 1992). The findings are in accordance with the biochemical experiments which showed that physiological activities of MLCK predominantly phosphorylate Ser19 and also with experiments in Ca^2+^-activated skinned fibres (Haeberle et al. 1988). Our MS/MS data and western blot analysis showing that pSer19 is the predominant m-phosphospecies of RLC in ET-1 contracted strips and that it is rapidly dephosphorylated during relaxation when intracellular [Ca^2+^], and hence, MLCK activity is low are consistent with these earlier reports. In addition, they are in keeping with reports that Ser19 phosphorylation is low in swine carotid arteries relaxed with forskolin (Meeks et al. 2008) as well as in α-toxin permeabilized mouse tail arteries relaxed with urocortin and cAMP (Lubomirov et al. 2006). As we have no indication for pThr9 phosphorylation shown to be involved in relaxation of vascular smooth muscle (Obara et al. 2008), our results suggest that relaxation is due to dephosphorylation of Ser19.

The surprising finding was, that our MS/MS analyses indicated that the m-phosphorylated RLC from 30 s relaxed preparations contained pThr18 (c.f. Fig. 2) and that western blots with phosphospecific antibodies suggest that m-phosphorylation of Thr18 increased during sustained relaxation (c.f. Fig. 3). We comprehensively identified RLC with a sequence coverage of up to 97 % and determined phosphorylation sites at Thr18 and Ser19 via LC/MS. Phosphate incorporation into Thr18 has been described before but typically as pThr18/pSer19 diphosphorylation generated by the action of Ca^2+^-independent RLC kinases such as integrin-linked kinase, ILK (Wilson et al. 2005), and ZIP kinase (Niiro and Ikebe 2001; reviewed in Walsh 2011). Compared to vascular smooth muscle (Weber et al. 1999, c.f. Fig. 3c, lane 1), diphosphorylation of intestinal smooth muscle by Ca^2+^-independent RLC kinase was much lower (Ihara et al. 2009; Shcherbakova et al. 2010). In the EFS-relaxed fundus smooth muscle diphosphorylation was below the detection limit of the pThr18/pSer19 dual phosphorylation antibodies (c.f. Fig. 3c, lanes 2–4). Notably, we also did not detect the corresponding diphosphorylated peptide species in the m-phosphorylated spot by LC/MS providing a high mass accuracy of 1–3 ppm in MS survey scans. Hence, we argue that pThr18 phosphorylation is not due to a contamination with diphosphorylated RLC. Taken together our MS/MS and western blot data indicate that the phosphorylation rebound seen in 2D-PAGE is a consequence of intricate phosphorylation events taking place at these two amino acid residues. Since western blot analyses suggest that phosphorylation of Ser19 declines to about 20 % of the ET-1 value, we propose that Thr18 m-phosphorylation largely accounts for the m-phosphorylation rebound. It is not clear at present whether the residual Ser19 phosphorylation is due to residual activation of MLCK or Rho kinase which is known to phosphorylate Ser19 Ca^2+^-independently and with higher efficacy than Thr18 (Kureishi et al. 1995).

There are several limitations of our study: (i) we do not know whether phosphorylation of Ser1 and 2 contribute to m-phosphorylation, (ii) the semiquantitative nature of western blots in particular with the pThr18 phosphospecific antibody, and (iii) that the phosphospecific antibodies only allow to determine the relative intensity changes between different treatments for a given antibody but not to reliably calculate the ratio between m-pThr18 and m-pSer19 during ET-1 and EFS. For instance, if one takes into account the relative changes of the immunoreactive signals during EFS, then the maximally ~1.7 fold increase in Thr18 would call for a ratio of pSer to pThr ~1:1 in ET-1 treated preparations which is clearly at variance with our MS/MS data and with previous investigations which showed that Thr18 was the minor phosphorylated site and reported a pSer19/pThr18 ratio of 6:1 in ^32^P labelled smooth muscle tissue (Csabina et al. 1986). We note, however, that m-pThr18 was observed in phosphopeptide maps and amino acid analysis of gizzard myofibrils phosphorylated in the absence of Ca^2+^ (Weber et al. 1999). As mentioned, quantitative information regarding the different RLC phosphospecies is very limited in intact smooth muscle and it might be argued that the diverging results are due to the fact that investigations with ^32^P labelled tissue can detect only phosphorylation turnover, whereas non-radioactive methods like LC/MS/MS and western blots with phosphospecific antibodies detect relative changes induced by different agonists and also permanent phosphorylations but not phosphorylation turnover. Thus, phosphoamino acid analysis with ^32^P may not detect pThr18 if it is not or only very slowly turning over compared with pSer19. Nevertheless our MS/MS data also do not support such a high level of Thr18 phosphorylation in ET-1 treated preparations, whereas they support the increase in pThr18 in EFS-treated preparations. Thus, while we cannot give at present the stoichiometries for the different phosphospecies in m-phosphorylated RLC, our results support the idea that there is a redistribution during maintained relaxation in favour of pThr18. In this context, it is of interest that phosphorylation of Ser19 is not a prerequisite for Thr18 phosphorylation (Bresnick et al. 1995).

Our findings raise the question as to which kinase(s) or phosphatase(s) are responsible for m-phosphorylation of Thr18 at low intracellular [Ca^2+^] and whether EFS activates such a kinase. Of the Ca^2+^-independent RLC kinases currently in question, ILK rather than ZIP kinase appears to be an attractive candidate. This is because in Ca^2+^-sensitized ileal smooth muscle ILK was proposed to be downstream of PKC (Ihara et al. 2009), and PKC may still be active during EFS-induced relaxation because of the continued presence of ET-1. In contrast, Ca^2+^-sensitization attributed to ZIP kinase was not blocked by inhibition of PKC in vascular smooth muscle (Choi et al. 2011). However, these Ca^2+^-independent RLC kinases phosphorylate Thr18 and Ser19 with equal efficiencies whereas our results ask for a kinase with a preference for Thr18 during cyclic nucleotide-induced relaxation. Another possibility would be that Ser19 is preferentially dephosphorylated in the relaxed preparations. However, although several phosphatases have been isolated from smooth muscle tissues, there is at present no evidence for a differential dephosphorylation of Ser19 over Thr18 (Ikebe et al. 1986; Feng et al. 1999). Thus, the mechanism that accounts for the shift in the phosphorylated residues is currently unclear.

In recent years, pThr18 phosphorylation has gained attention as an index of the action of Ca^2+^-independent kinases and was mainly considered to be present in diphosphorylated RLC. Whereas diphosphorylated myosin enhanced actin-activated MgATPase activity in solution (Ikebe and Hartshorne, 1985), it had no additional effect on force in skinned fibres (Haeberle et al. 1988). In contrast, the actin-activated myosin ATPase activity of myosin m-phosphorylated on Thr18 is ~15-fold lower than that phosphorylated on Ser19 (Bresnick et al. 1995). Surprisingly, Thr18 m-phosphorylated myosin was able to move actin filaments in the in vitro motility assay similar to that m-phosphorylated on Ser19 or both Thr18/Ser19. We are not aware of an investigation of the effect of Thr18 m-phosphorylation on force. However, our results suggest that pThr18 m-phosphorylated myosin does not support force. Exchanging endogenous RLC with m-phosphorylated at Thr18 in skinned fibres should help to resolve this question. Thus, it is also not known whether m-phosphorylation of this site influences relaxation or whether the phosphorylation rebound is a paraphenomenon of high NO activation. As pThr18 m-phosphorylated myosin is capable for dimerization and filament formation, it is tempting to speculate that it may help to stabilize myosin filaments at low levels of RLC phosphorylation at Ser 19. Further studies are required to assess the functional relevance of pThr18 m-phosphorylation during relaxation.

Assuming that pSer19 regulates attachment of crossbridges and that the remaining phosphorylation of Ser19 is below the threshold for activation of contraction, dephosphorylation of Ser19 is sufficient to induced relaxation and no additional regulatory mechanisms are required for switching off the contractile machinery. Of note, RLC phosphorylation was high before starting relaxation. However, relaxation by cAMP/cGMP has also been induced at low levels of RLC phosphorylation, i.e. during the latch state (Miller et al. 1983; Gerthoffer et al. 1984; Fischer and Pfitzer 1989; Khromov et al. 1995). In this situation, additional mechanisms may still be necessary which either increase the net detachment rate of dephosphorylated crossbridges or inhibit the cooperative reattachment of dephosphorylated crossbridges (Albrecht et al. 1997, Malmqvist et al. 1997; reviewed in Kim et al. 2008).

In conclusion, the initial phase of cGMP/cAMP-mediated relaxation of gastric fundus smooth muscle induced by the release of inhibitory neurotransmitters from intrinsic neurons was associated with a decline in m-phosphorylation of RLC. However, during the sustained phase of relaxation phosphorylation rebound to the values before starting relaxation. This suggested that stress is uncoupled from RLC dephosphorylation as has been observed in vascular smooth muscle relaxed by NO (Rembold et al. 2001). Determining the sites phosphorylated during the sustained phase of relaxation revealed that the phosphorylation rebound is mainly due to m-phosphorylation of Thr18 whereas Ser19 is dephosphorylated consistent with current concepts of the regulation of smooth muscle contraction. Finally, the combination of Phos-tag gels with western blotting should rapidly advance our understanding of the contribution of different phosphospecies of RLC to the regulation of smooth muscle function.

## Abbreviations

Tris: 2-Amino-2-(hydroxymethyl)-propan-1,3-diol
HEPES: *N*-(2-hydroxyethyl)piperazine-*N*′-(2-ethanesulfonic acid)
DTT: Dithiothreitol
RLC: 20 kDa regulatory myosin light chain
m-phosphorylation: Monophosphorylation
ET-1: Endothelin 1
EDTA: Ethylenediaminetetraacetic acid
EFS: Electrical field stimulation
l-NAME: *N*_ω_-nitro-l-arginine methylester hydrochloride
MLCK: Myosin light chain kinase
MLCP: Myosin light chain phosphatase
